# Current status and development of neutron radiation for biophysical applications in Colombia

**DOI:** 10.1007/s12551-023-01079-0

**Published:** 2023-07-27

**Authors:** J. Alfonso Leyva, Edwin Munévar

**Affiliations:** 1 Departamento de Física, Pontificia Universidad Javeriana, Cra. 7 No 40-62, Bogotá, 110231 Cundinamarca Colombia; 2 Proyecto Curricular de Licenciatura en Física, Universidad Distrital Francisco José de Caldas, Cra. 3 No 26A-40, Bogotá, 110311 Cundinamarca Colombia

**Keywords:** Neutron radiation, Neutron capture therapy, Boron, Geant4, Medical physics

## Abstract

In Colombia, medical physics started formally about 3 decades ago. Two master’s programs in medical physics initiated activities at two different universities. In particular, the master’s program at the Pontificia Universidad Javeriana has been underway since 2012, and taking into account its projections, a team was established in 2015 in collaboration with the Universidad Distrital Francisco José de Caldas to conduct basic research on cancer treatment using neutron capture therapy (NCT). The primary goal of our initiative is to create the infrastructure required to adapt new technologies in our universities in the future. The long-term objective is to use neutron radiation to study not only NCT but also biomolecules, membranes, and materials. This will require the commissioning of an actual nuclear facility. Our group has been exclusively focused on carrying out calculations with GEANT4 because of its characteristics as open-source software, its accessibility, and its ample worldwide use and validation in the particle physics, nuclear physics, and medical physics communities. In this work, we present some results of our preliminary design for the ion accelerator column of a compact neutron generator. Also, we present the characterization of the kinematical and dose distributions of boron neutron capture processes using Geant4.

## Introduction

Since 1932, when the cyclotron was used to produce artificially created radioactive isotopes for medical applications in cancer treatment (Lawrence and Livingston (1932)), particle accelerators have transformed from high-tech experimental facilities, exclusive to laboratories for nuclear physics and elementary particles, mainly, to research and development tools in other areas of science and technology. Today, particle accelerators are in common use in biophysics laboratories, materials science, radiochemistry, semiconductors and microcircuits, radiotherapy, security, and a huge number of industrial applications. The development and dissemination of accelerators have been possible thanks to advances in the physics of charged particle beams, microwave technologies, the use of new materials, and control and processing methods, among others (Sauerwein et al. (2012)).

With the discovery of the neutron (Chadwick (1932)), research in nuclear physics and its applications was developed. Considerable efforts were focused on the so-called neutron capture. This type of nuclear reaction was described as early as 1935 (Taylor and Goldhaber (1935)). In 1936, the biological effects of the nuclear neutron capture reaction were specified, and its potential for use as a cancer therapy was suggested (Locher (1936)). This procedure was later called neutron capture therapy (NCT).

NCT combines the introduction of small amounts of a neutron absorber such as $^{10}\text {B}, \,^{157}\text {Gd}$, and $\,^7\text {Li}$ that are stored at the cellular level, subsequently being bombarded with low-energy neutrons, which trigger a reaction that releases ionizing radiation in the regions of greatest interest, thus providing a selective way to cause damage to tumor cells while at the same time minimizing the lethal effects on surrounding healthy tissue (Sauerwein et al. (2012)).

In particular, when NCT uses boron $(^{10}\text {B})$ as an absorbing medium, it is called boron neutron capture therapy (BNCT). The nuclear reaction that takes place is given by the following:

1 $$\begin{aligned}{}[^1_0\text {n} ]+[^{10}_5\text {B}]\rightarrow [^{11}_5\text {B}]^* \rightarrow {\left\{ \begin{array}{ll} \quad [^4_2\text {He}] \quad + &{} [{^7_0\text {Li}}] \quad + \quad \text {2.79 MeV} \qquad (6.1 \% ) \\ \quad [^4_2\text {He}] \quad + &{} \underbrace{[^7_3\text {Li}]^*} \quad + \quad \text {2.31 MeV} \qquad (93.9 \% ) \\ &{} [^7_3\text {Li}]^* \rightarrow [^7_3\text {Li}] + \gamma \end{array}\right. } \end{aligned}$$

The main decay channel releases gamma rays $\gamma \,(0.478$ $\text {MeV})$, the so-called $\alpha$ particles, i.e., $^4\text {He} \,(1.47 \,\text {MeV})$ and lithium ions $^7\text {Li} \,(0.84 \,\text {MeV})$ within the cell tissue. This channel has a branching ratio of $93.9 \%$. The secondary channel has a branching ratio of $6.1 \%$ and releases only $\alpha$ particles [$^4\text {He} \,(1.78 \,\text {MeV})$] and lithium ions $^7\text {Li} \,(1.01 \,\text {MeV})$ without the production of gamma rays. These nuclear reactions yield high linear energy transfer (LET) $^4\text {He}$ and $^7\text {Li}$ with typical average values of 100–200 keV/$\mu$m and 20–40 keV/$\mu$m, respectively. Compared to the average cell diameter in the human body ($\approx 50 \,\mu$m), these reaction products are released within average path lengths of roughly 5–9  $\mu$m. Consequently, the destructive effects are confined to the boron-containing cell, while adjacent, healthy cells are, in theory, unaffected.

The efficiency of BNCT is, however, limited by the fraction of the deposited energy that cannot be controlled or targeted specifically. The impact of the therapy is then not exclusive to the targeted tumor cells, but can extend to the adjacent healthy cells as well. As a consequence, such an occurrence could potentially lead to unfavorable and harmful impacts on the surrounding healthy tissue. Therefore, to achieve a high efficiency in BNCT, a boron concentration of the order of $10^9$ atoms per cell is required, then the boron concentration should be of approximately 35 $\mu g$ per gram (35 ppm) of tumor tissue, while for healthy tissue, the concentration should not exceed 5 $\mu g$ per gram (5 ppm) of tissue (Hanaoka et al. (2014); Hosmane et al. (2012)). High concentration can be achieved by either using a boron-containing compound that selectively accumulates in the cancer cells or by directly injecting a boron compound into the tumor. The accurate measurement of $^{10}$B concentration in both tumor and normal cells plays a crucial role for determining the maximum irradiation time during BNCT. Insufficient irradiation time may result in inadequate $^{10}$B concentration within the tumor cells, leading to ineffective destruction of cancerous cells. Conversely, with a prolonged irradiation time, the dose delivered to healthy cells could surpass the critical threshold, resulting in adverse effects. By achieving this balance between tumor damage and healthy tissue preservation, BNCT has the potential to treat specific types of cancer.

**Fig. 1:**
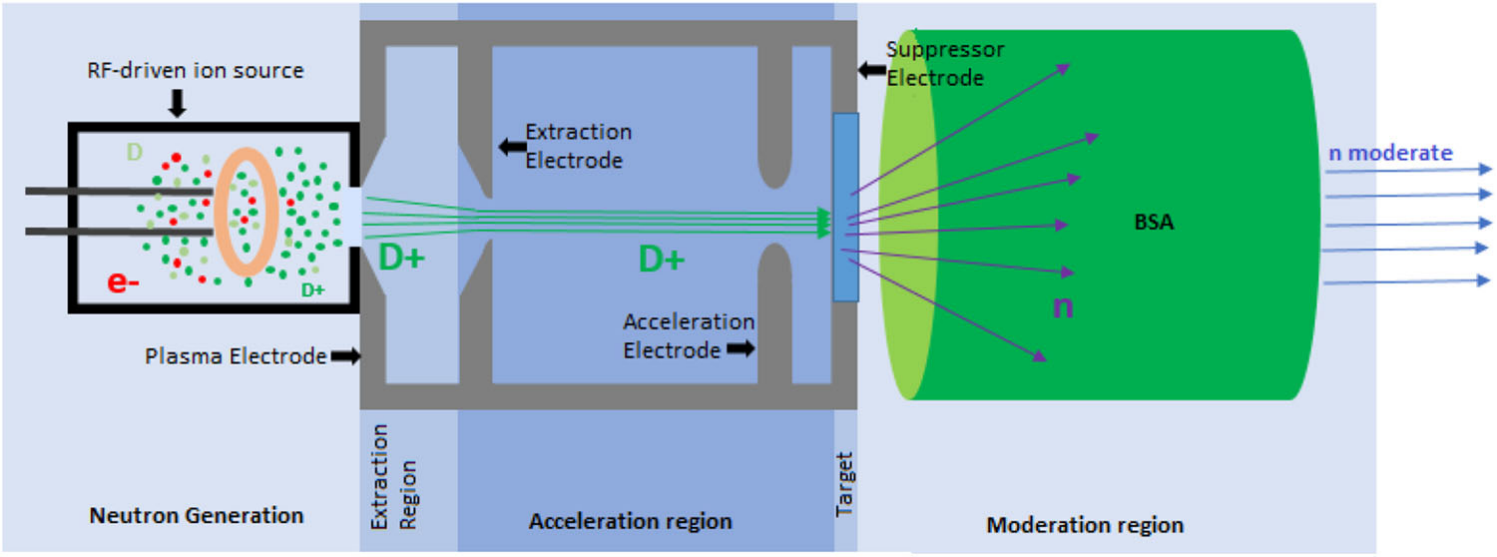
Neutron compact generator diagram. The main modules are shown from left to right: ion source, extraction region, accelerator column, target, and the beam-shaping assembly (BSA) or moderator

The BNCT development was primarily addressed to brain cancer, i.e., glioblastoma multiforme (GBM). This type of tumor is considered one of the most aggressive cancers and can rarely be surgically removed (Kankaanranta et al. (2012)). It is characterized by its aggressive proliferation and extensive invasion of healthy tissue before any symptoms appear. The rate of sterilization or destruction of these tumors is higher compared to other techniques, such as radiotherapy by X-rays.

The success of BNCT as a treatment for this class of tumors depends on several factors: an adequate concentration of $^{10}\text {B}$ in the tumor cells, differential uptake of boron in tumor cells and in healthy cells, and sufficient fluence of thermal neutrons in the tumor region (Moghaddasi and Bezak (2017)).

For these reasons, current research focuses on the development of medications capable of transporting boron to cancerous tissues in a differential way, as well as neutron sources that pose no threat to a medical facility. Implementation of compact neutron generators is currently of considerable interest.

Finally, we have established collaborations with research groups around the world. That is the case for the group of Dr. K. Leung at the Lawrence Berkeley National Lab (LBNL) at the University of California, Berkeley, in the USA, for the design and construction of a compact neutron accelerator (Verbeke (2000); Verbeke et al. (2000)).

## Compact neutron generator

Until 2009, most of the research for therapeutic treatments with BNCT was carried out using nuclear reactors that generate significant fluxes of neutrons through chain reactions of nuclear fission (the thermal reactors used for BNCT are typically research or test reactors, i.e., TRIGA model). The results of these studies are quite promising; however, the difficulties involved in having a nuclear reactor near a hospital facility prevent further research into neutron therapy (Kim and Kim (2009)). The generation of suitable neutron beams for BNCT near a hospital facility is a technological challenge. These slow neutron beams must be obtained in a safe and stable manner. Currently, the main advances have been made in the USA, Japan, China, Russia, the UK, Italy, Israel, and Argentina (Kim and Kim (2009); Kreiner et al. (2016)). After almost 60 years of research and development on BNCT with nuclear reactors, engineering efforts were focused on the development of a compact, hospital-based proton accelerator system for BNCT. Currently, a functional prototype of compact high intensity neutron generators is technologically available (Bavarnegin et al. (2019)).

Essentially, these compact neutron generators accelerate charged particles, i.e., D$^+$ ions, which then collide with a deuterium ($^2$H) or tritium ($^3$H) rich target (titanium embedded with deuterium or tritium), generating the neutrons through a mechanism of nuclear fusion between the doped target ions (Leung et al. (2004)). With a $^2$H target, neutrons (n) are emitted with a kinetic energy of 2.45 MeV according to the reaction:

2 $$\begin{aligned} \textrm{D}^+ + \mathrm {^2H} \rightarrow \mathrm {^3He} + \textrm{n} + \mathrm {(Q=3.27\,\, MeV)} \end{aligned}$$

where the $^3$He is produced with a kinetic energy of 0.82 MeV. With a $^3$H target, the neutrons are emitted with a kinetic energy of 14.05 MeV according to the following:

3 $$\begin{aligned} \textrm{D}^+ + \mathrm {^3H} \rightarrow \mathrm {^4He} + \textrm{n} + \mathrm {(Q=17.6\,\, MeV)} \end{aligned}$$

and the $^4$He is produced with a kinetic energy of 3.54 MeV.

The compact neutron generator is mainly composed of the following modules: the ion source, extraction region, region of acceleration or accelerator column, a target, and the beam-shaping assembly (BSA), as shown in Fig. 1.

The prototype for the compact neutron generator that we are working on is based on what has already developed at LBNL (University of California, Berkeley) in the group of Dr. Leung (Verbeke (2000); Verbeke et al. (2000)). The specifications of this design are shown in Table 1.

As for the ion source, which ionizes deuterium at low pressure ($5\, \text {mTorr}$) with radio frequencies (operating frequency of $13.5\, \text {MHz}$) and a temperature of the plasma of $1\, \text {eV}$, it produces deuterons that will then be extracted to the accelerator column, where they will obtain the necessary kinetic energy to collide with the deuterium-rich target and thus generate neutrons.

The initial proposed design parameters consider a deuteron energy of 0.2 to 0.5 MeV, despite the fact that $D-D$ reaction cross section has a peak between 0.7 and 0.8 MeV, obtaining neutrons with an energy of 2.4 MeV. It is to be considered that for the reaction (2), in general, the neutron energy can be calculated from four-momentum conservation and is given by the expression (Cifuentes (2019)):

4 $$\begin{aligned} E_{n}^{1/2}\!= & {} \!\frac{{\left( {m}_{d}{m}_{n}{E}_{d}\right) }^{1/2}}{{m}_{B}\!+\!{m}_{n}}cos\left( \theta \right) \nonumber \\{} & {} \!+\! \frac{{\left\{ {m}_{d}{m}_{n}{E}_{d}{cos}^{2}\theta \!+\!\left( {m}_{B}{m}_{n}\right) \left[ {m}_{B}Q\!+\!\left( {m}_{B}\!-\!{m}_{d}\right) {E}_{d}\right] \right\} }^{1/2}}{{m}_{B}\!+\!{m}_{n}} \end{aligned}$$

where ${E}_{n}$ is the neutron kinetic energy, ${m}_{d}$ is the deuteron mass, ${m}_{n}$ is the neutron mass, ${E}_{d}$ is the deuteron kinetic energy, and ${m}_{B}$ is the mass of the product $B=$ $^{3}He$. $\theta$ is the neutron’s emission angle.

**Table 1:** Design characteristics of the prototype for the compact neutron generator, using a titanium target embedded with deuterium

Element	Value
Neutron source	$^{2}\text {H} \left( d,n\right) ^3\text {He}$
Fluence	$1 \times 10^{10} \frac{n}{s}$
Flux	$1 \times 10^{9} \frac{n}{cm^{2} s}$
Ion type	$D^{+}$
Current density	$5 \frac{mA}{cm^{2}}$
Deuteron energy	0.2 MeV to 0.5 MeV
Deuteron current	1500 mA
Neutron energy	1.8 MeV to 3.5 MeV
Target	Titanium embedded with deuterium

We decided to start with the accelerator column, also in order to acquire experience and sufficient acknowledgement to be able to develop and build the other modules. Additionally, we can strengthen and build collaborations with other international groups and apply for funding.

### Results

We present preliminary calculations for the accelerator column. Basically, we need to solve the Poisson and Laplace equations with the corresponding boundary conditions to solve the electric field $\textbf{E}$ (Leung et al. (1998); Verbeke et al. (1999)).

**Fig. 2:**
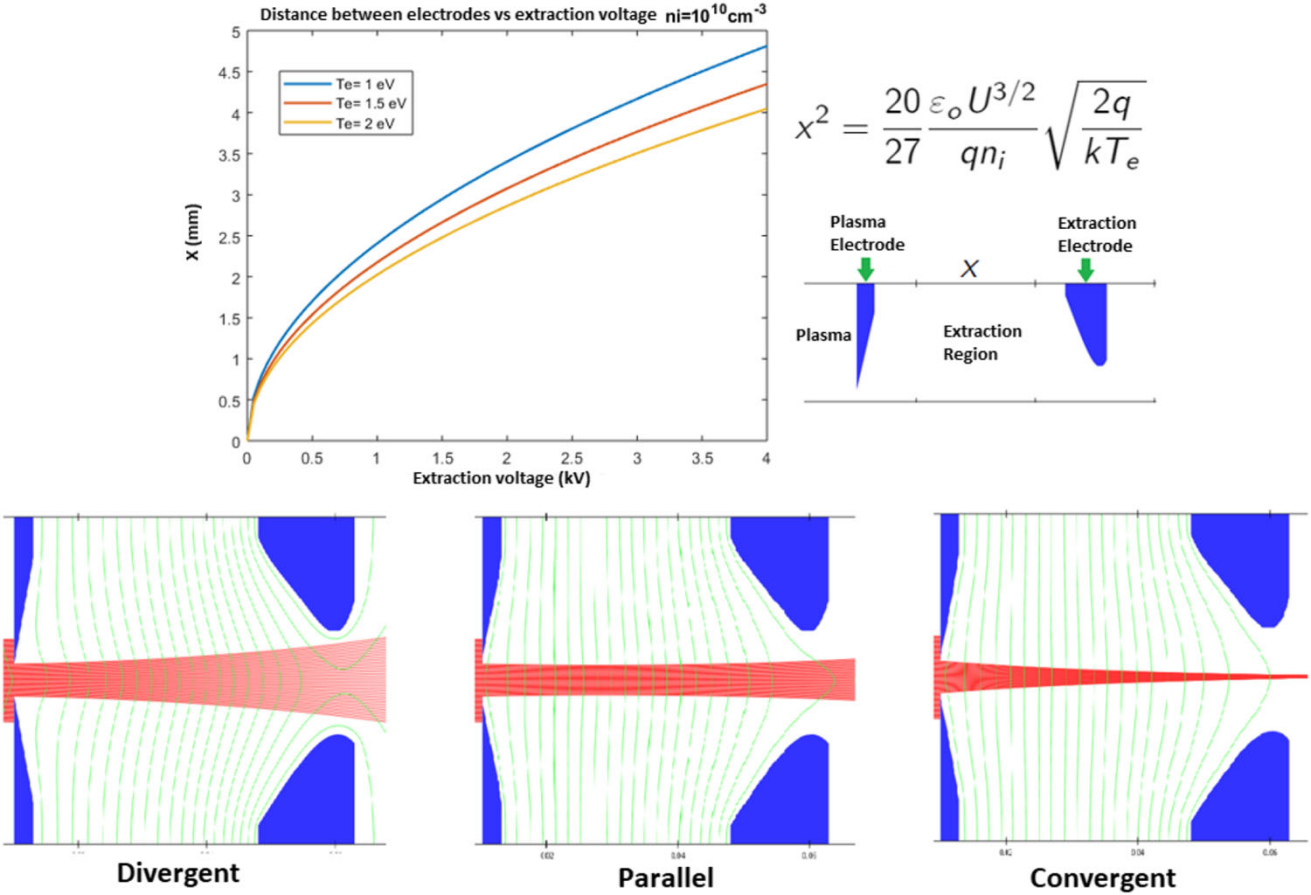
Variation of the distance between the plasma electrode and the extraction electrode with respect to extraction voltage. This plot follows the equation presented in the upper right corner. The divergent, parallel, and convergent ion beam depends critically on the distance between the plasma electrode and the extraction electrode, as well as the extraction voltage

Figure 2 shows the distance *x* between the plasma and extraction electrodes as a function of the extraction voltage. By combining the Child-Langmuir and the Boltzmann equations, this relationship is obtained (Csikai (1987)). Clearly, the *x* distance has an effect on the ion beam profile. By adjusting this distance, a different ion beam configuration—parallel, convergent, or divergent—can be obtained. The choice of ion beam configuration depends on the specific requirements of the fusion experiment or device. Parallel beams allow for simple control and manipulation of the ion trajectories, ensuring a more efficient and homogeneous production of neutrons; convergent beams are typically employed to achieve higher fusion rates and higher neutron yields. Divergent beams are needed when one wants to reduce the beam power density on the target electrode. For instance, for D-D reactions, the deuterium atoms can be evaporated from the target surface if the beam power density is too high. As a result, the neutron yield will be reduced.

The extraction electrode separation distance *x* is therefore critical to guaranteeing a parallel ion beam for a given extraction voltage (characteristic of the extraction system) and a given ionic density (characteristic of the RF ion source). Upon calibration of these parameters, this profile of ion beam can be obtained.

## Boron Geant4 preliminary calculations

The intricate nature of the radiation field associated with BNCT has rendered Monte Carlo simulation an indispensable tool for addressing the low-energy neutron transportation problem. To simulate the passage of neutrons through matter, our group has relied primarily on Geant4 version 10.7  (Agostinelli (2003)), a software widely used in high-energy physics, nuclear physics, and medical physics, developed by an international collaboration of scientists and engineers from various institutions and distributed under the LGPL open-source license. Written in C++, its modular design and variety of implemented models enable it to be used for a vast array of applications, from simple particle tracking to complex simulations of detector systems and radiation therapy treatments.

In order to test the physics models employed in Geant4, we also used MCNP version 6.2 (Werner et al. (2018)), a general-purpose Monte Carlo code for simulating the behavior of particles in complex geometries. The testing consisted of comparing the kinematical and dose distributions (in water) from basic geometries and radiation sources using both Geant4 and MCNP. This allowed us to select the Geant4 physics packages whose results best matched those of MCNP.

Thus, we made use of electromagnetic and high-precision hadronic packages in our Geant4 simulation. In particular, for electromagnetic interactions, we used the G4EmStandardPhysics list, which provides a comprehensive treatment of electromagnetic interactions, including ionization, bremsstrahlung, multiple scattering, and positron annihilation, over a broad range of particle energies and materials. This physics list is based on the classical Bethe-Bloch theory for energy loss, the Goudsmit-Saunderson theory for multiple scattering, and the Bethe-Heitler model for bremsstrahlung processes. Hadronic interactions were implemented through the QGSP-BIC-HP physics list, which combines the QGSP model (Quark-Gluon String Precompound) and the Binary Cascade (BIC) model. For neutrons with energies below 20 MeV, it employs high-precision neutron models and improved cross-section data to account for processes such as elastic and inelastic scattering, capture, and fission. This physics list is dependent on the G4NDL database (Geant4 Collaboration (2021)).

In addition, Geant4 incorporates a model for thermal elastic scattering, which considers the impact of chemical bonds and crystal structures on the scattering process of neutrons by nuclei in materials such as water and polyethylene, particularly at low energies. The importance of this lies in the fact that the scattering characteristics of a substance may exhibit variability based on its molecular or crystalline configuration. For example, the scattering cross section of a water molecule differs from that of a single hydrogen or oxygen nucleus because the two nuclei in the molecule are bonded together. To account for the aforementioned, a special thermal scattering data set and model must be incorporated for neutron energies below 4 eV. This enhances the agreement between Geant4 and MCNP results (i.e., dose, fluence) at low neutron energies (Garny et al. (2009)).

**Fig. 3:**
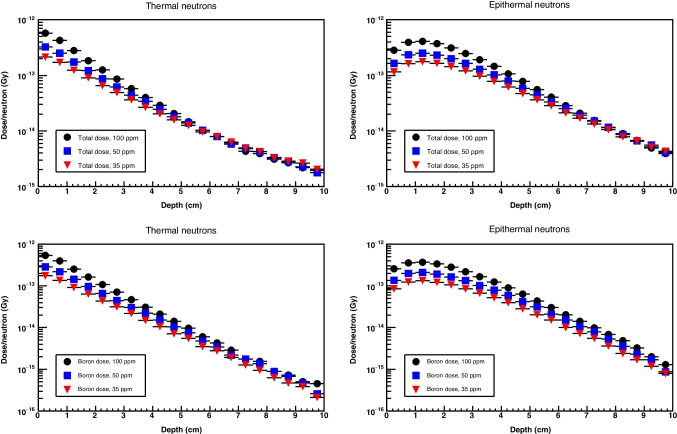
Total dose and boron dose normalized to the number of incident neutrons for tissue with concentrations of 35, 50, and 100 ppm of $^{10}$B: (top left) total dose for thermal neutrons of 0.025 eV, (top right) total dose for epithermal neutrons of 1.0 eV, (botton left) boron dose for thermal neutrons of 0.025 eV, and (bottom right) boron dose for epithermal neutrons of 1.0 eV

The simulations we have developed encompass regular-shaped targets composed of tissue with varying concentrations of $^{10}$B. The neutron sources consist of ideal beams assumed to be monodirectional and monoenergetic. Two different sources are independently implemented: a source of thermal neutrons with energy of 0.025 eV and a source of epithermal neutrons, defined as neutrons with energies between 0.4 eV and 10 keV. Since the probability of $^{10}$B capturing a neutron decreases with increasing neutron energy, low-energy (thermal) neutrons have a higher probability of capture than high-energy (epithermal) neutrons, resulting in a higher yield for the boron neutron capture reaction. In contrast, epithermal neutrons have a longer range in tissue and can penetrate deeper into the human body than thermal neutrons. This characteristic renders epithermal neutrons more appropriate for the treatment of tumors that are located at greater depths. To assess the impact of both neutron sources on the dose distribution, simulations were conducted with each.

### Results

It can be observed that the radiation field in BNCT is comprised not only of thermal (or epithermal) neutrons but also of $\alpha$ and $^7$Li from $^{10}$B neutron capture reactions, photons from $^1$H(n, $\gamma$)$^2$H reactions, protons from $^1$H(n, n$^\prime$)$^1$H and $^{14}$N(n, p)$^{14}$C reactions, as well as $^{14}$C, and other heavy charged particles. When estimating the absorbed dose, it is necessary to differentiate between dose associated only with $^{10}$B, i.e., dose from $\alpha$ particles and $^7$Li nuclei (termed boron dose) and dose resulting from reactions with tissue constituents, i.e., dose mainly from $\gamma$, p, and $^{14}$C (termed non-boron dose).

The simulation is based on a 10-cm-long, 5-cm-wide cylinder containing tissue with concentrations of 35, 50, and 100 ppm of $^{10}$B. For thermal neutrons, an energy of 0.025 eV was used. For epithermal neutrons, energies of 1 eV, 10 eV, 100 eV, 1 keV, and 10 keV were implemented. Figure 3 shows the total dose and the boron dose calculated in Geant4 by using thermal neutrons and the lower-energy epithermal neutrons (1 eV). As can be seen, the increasing concentration yields a higher dose only up to about 5 cm. Beyond that, there is virtually no change in both the total dose and the boron dose. In addition, the maximal boron dose for thermal and epithermal neutrons occur at 0.5 and 1.5 cm inside the patient’s body, respectively. Both decrease by approximately two orders of magnitude beyond 8 cm. With higher-energy epithermal neutrons (10 eV - 10 keV), the maximum boron dose occurs at a depth of roughly 3.0 cm for 10 keV, and the maximum depth before the dose decreases by two orders of magnitude is about 10 cm. These results indicate that BNCT cannot provide effective treatment for deep-seated tumors located at a distance of 10 cm or longer.

## Concluding remarks

In this work, we present some of the achievements of the NCT initiative in Colombia. In the first place, the simulations of the nuclear reaction via Geant4 are completely feasible, despite some difficulties due to the completeness of the physics libraries regarding the required energy scale of the different intermediate and final states. Secondly, the preliminary design of the accelerator column shows a high degree of similarity with respect to the developments at the Lawrence Berkeley National Laboratory (LBNL) at the University of California, Berkeley, in the USA. At this point, it is of great relevance to mention that beyond the similarities between both designs, there are differences, mainly the voltages of acceleration and extraction, which are expected to increase the neutron fluence. Finally, and of great relevance for our young NTC community, in the last years, we have had 8 graduate masters students (in medical physics and engineering masters programs) and 10 physics bachelors, slowly generating the necessary critical mass of researchers for the future. A group of the master students have focused their research in a first approach to the compact neutron generator and some of the problems associated with it, writing their thesis on the following: Preliminary evaluation of the acceleration of D in a compact high-flux D-D neutron generator (Cifuentes (2019)), Preliminary dose calculation in boron neutron capture therapy using removal diffusion theory (Cruz (2019)), Preliminary evaluation of neutron moderation in a high flux compact D-D generator (Velásquez (2019)), Preliminary algorithm for calculating neutron flux and dose for neutron capture therapy by implementing the Boltzmann lattice method (Luna (2019)), and Computational approach to sterilization of the human amniotic membrane the via Geant4 using ionizing radiation (Pabón (2017)). The undergrade students have worked on the following: Characterization of the interaction of neutral ionizing radiation at the cellular level using a computational simulation with Geant4 (Dominguez (2021)), Computational approximation through Geant4 of the nuclear reaction of neutron capture by hydrogen present in biological cells (Rodríguez (2021)), Computational evaluation of the structural dynamics of a biomolecule, after its interaction with charged particles (Rubio (2020)), Computational study of the structural dynamics of DNA, after its interaction with ionizing radiation (Salazar (2020)), Characterization of the neutron capture reaction by $^{16}$O (Sanabria (2022)), Computational evaluation of the microbiological load in biomembranes under treatment with ionizing radiation (Páez (2023)), Cancer: a biophysical look through the cell membrane (Ortega (2021)), Computational approximation via Geant4 of the interaction of $^7$Li from neutron capture in biological cells (Medina (2019)), and Computational approximation of the interaction of gamma radiation from neutron capture in biomolecules through Geant4 (Tellez (2019)). In the last years, we have been building the technical-scientific infrastructure necessary to address the problems of the generation and application of neutron radiation to biophysical systems in our country.

## Data Availability

The data that support the findings of this study are available from the corresponding author upon reasonable request.

## Code Availability

The code associated with this study is available from the corresponding author upon reasonable request.
